# Conceptualizing citizenship in dementia: A scoping review of the literature

**DOI:** 10.1177/14713012221111014

**Published:** 2022-06-29

**Authors:** Deborah O’Connor, Mariko Sakamoto, Kishore Seetharaman, Habib Chaudhury, Alison Phinney

**Affiliations:** School of Social Work, 8166 University of British Columbia, Vancouver, BC, Canada; Centre for Research on Personhood in Dementia, 8166 University of British Columbia, Vancouver, BC, Canada; Department of Gerontology, 1763 Simon Fraser University, Vancouver, BC, Canada; School of Nursing, 8166 University of British Columbia, Vancouver, BC, Canada

**Keywords:** Dementia, citizenship, scoping review, human rights, agency and autonomy

## Abstract

Citizenship has provided an important conceptual framework in dementia research and practice over the past fifteen years. To date, there has been no attempt to synthesize the multiple perspectives that have arisen in this literature. The purpose of this paper is to explore, reflect on, and contrast, the key concepts and trends in the citizenship discourse as it relates to people with dementia. Using a scoping review methodology, forty-nine articles were identified for review. Despite the use of different descriptors, thematic analysis revealed four core themes underpinning citizenship discourse: 1) the relationality of citizenship; 2) facilitated agency and autonomy; 3) attention to stigma, discrimination and exclusion; and 4) recognition of the possibilities of identity and growth. Overall, this scoping review found a major emphasis on expanding definitions of agency and autonomy to render citizenship unconditional and inclusive of the diverse life experiences of people living with dementia. Notably, there is recognition that a more intersectional lens for embedding the subjective experience within a broader socio-political context is needed. Whilst the adoption of a citizenship lens in dementia research and practice has had real-world implications for policy and research, its exploration and use continue to be led by academics, highlighting the importance that future research involve input form people with dementia.

## Introduction

Historically, research and practice have framed dementia – or neurocognitive disorder (NCD) - through a deficit-focused biomedical approach, which assumes that the primary experience of people living with dementia could only be understood in relation to neuro-cognitive degeneration and loss. Recognizing the limitations of this dominant approach for understanding, a personhood lens was introduced a few decades ago through the work of pioneers such as Kitwood (1989; 1997) and Sabat and Harré (1992). This lens meaningfully expanded the scope of understanding the dementia experience beyond neuro-cognitive loss to take into account the influences of people’s personal histories and relationships. This relational lens posits that the lived experience of people with dementia is shaped by how they interact with their interpersonal environment, noting particularly the negative impact of a societal lens that presumes incapability and defines the person by the diagnosis of dementia.

Despite its importance and impact on broadening how people with dementia are understood and treated, the personhood lens has been critiqued for failing to recognize people with dementia as active agents with existing rights, and to adequately account for the power imbalances in society that cause stigma and discrimination. Pioneering work by Bartlett and O’Connor (2007) used the language of social citizenship to advocate for integrating a more critical and socio-political lens to understand the lived experience of dementia. These ideas were then more formally developed in their 2010 book, *Broadening the Dementia Debate: Toward Social Citizenship,* which included a working definition and conceptual framework of ‘social citizenship’ to provide a common foundation and direction for the advancement of research and practice using this approach. They define social citizenship as ʻa relationship, practice or status, in which a person with dementia is entitled to experience freedom from discrimination, and to have opportunities to grow and participate in life to the fullest extent possible. It involves justice, recognition of social positions and the upholding of personhood, rights, and a fluid degree of responsibility for shaping events at a personal and societal levelʼ (p. 37).

Whilst this framework provides a core foundation for considering a citizenship-based lens in dementia studies, multiple theoretical perspectives in subsequent research have now arisen. To date, there has yet to be an attempt to compare and synthesize this body of knowledge. In particular, it is unclear how the conceptual ideas underpinning the different citizenship approaches extend and/or challenge one another. The purpose of this paper is to review the literature on citizenship of people living with dementia to explore, reflect on, and contrast, the key concepts and trends in the citizenship discourse. Synthesizing and integrating the multiple perspectives that have emerged through conceptual and empirical development in this area will help make explicit the core assumptions and values that underpin this approach, whilst simultaneously identifying tensions and discrepancies that exist among the different perspectives. These observations will be useful in articulating this approach, setting priorities, and charting the direction for future development of this body of knowledge.

## Methods

A scoping review methodology was adopted to map the literature on citizenship and dementia and extract themes that represent the body of knowledge in this area. The steps involved in this review process included: (a) identifying the research question; (b) identifying studies pertaining to the research question; (c) screening and selecting studies; (d) charting data, and (e) collating and summarizing the results (Arksey & O’Malley, 2005). Scoping reviews are ideal for a quick and broad examination of the range of literature on a given topic.

The research question: ʻHow has citizenship been defined and applied in dementia research?ʼ guided this review. The search string with Boolean operators used to conduct the search for articles in this paper were *(dementia* OR Alzheimer*) AND (citizen* OR ʻ*human rights*ʼ OR *agency* OR *discrimination)*. The addition of the keywords ‘agency’ and ‘discrimination’ was intended to produce a more expansive range of articles in the initial pool for screening. The databases accessed to search for articles were Academic Search Premier, AgeLine, CINAHL Complete, Global Health, Google Scholar, Medline with Full Text, PsycINFO, Social Sciences with Full Text, JSTOR, and Web of Science.

The following inclusion criteria were followed to screen the articles:

• publication type: peer-reviewed journal article including research studies or discussion papers;
• publication date range: 2007–2019;
• language: English;
• topic of discussion or study population: persons living with dementia, and conceptualization and/or application of citizenship in the context of dementia care.

**Figure 1.**
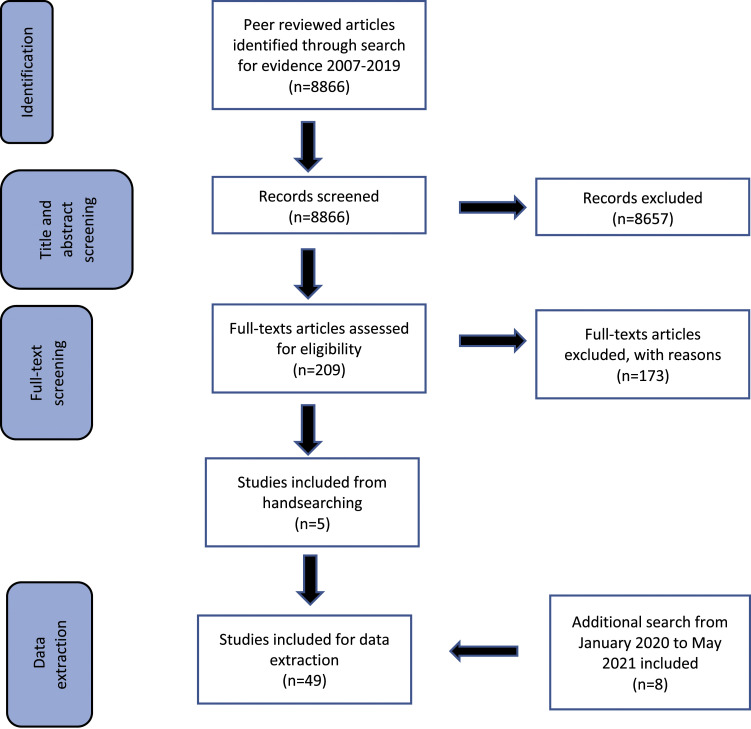
Step-wise process of screening articles.

Figure 1 outlines the process of screening of articles. Firstly, the titles and abstracts of the 8866 search results were screened according to the inclusion criteria. Following this first stage of screening, 8657 results were eliminated due to the failure to meet inclusion criteria, particularly, the lack of fulfilment of focus on people living with dementia and/or the adoption of a citizenship framework. The full-text articles of the remaining 209 items were then screened based on the same inclusion criteria. At this stage, 173 articles were eliminated due to their lack of conceptualization or emphasis on citizenship of people living with dementia (e.g., not adopting a citizenship approach to understanding and conceptualizing the dementia experience or briefly mentioning extant perspectives on citizenship but not applying them in the study context). Consistent with scoping review parameters (Arksey & O’Malley, 2005), additional papers from the reference lists of included articles were hand-searched which fielded an additional five articles. Forty-one articles were finalized for in-depth review at this stage.

The key findings, propositions, and arguments from these items were extracted, focusing on the language being used with respect to citizenship and dementia, as well as the main components of citizenship discussed in the articles. The extracted data were closely examined for common themes, which were then identified and used to organize and tabulate the data into a chart. The analysis of the extracted data to derive key themes was loosely based on the principles of thematic analysis (Braun & Clarke, 2006), however, no formal qualitative coding was conducted.

Following the analysis of the 41 items, published between 2007 and 2019 (original search), a subsequent search was conducted using the aforementioned keywords and databases for articles published up to May 2021. This second stage search resulted in eight additional articles and was done as both an update and to see how new and emerging directions in research aligned with the interpretation of key themes and concepts thus far – it helped to validate and tighten the existing analysis. This resulted in a final total of 49 items included in the review. Overall, this includes 21 conceptual papers, one scoping review and 27 research studies - all of which utilized qualitative methods, ranging from ethnographic observations and interviews to focus groups. This set of papers, with only three exceptions – two research papers (O’Connor et al., 2018; Wiersma et al., 2016) and one conceptual paper (Mann, 2020)- did not explicitly include co-authors who lived with dementia.

## Results

Whilst the articles all adopted the language of citizenship as a general concept, authors often used specific terms to label their approach, for example narrative citizenship (Baldwin, 2008), social citizenship (Bartlett & O’Connor, 2007, 2010), relational citizenship (Kontos et al., 2016), micro-citizenship (Baldwin & Greason, 2016; Mitra & Schicktanz, 2020), and active citizenship (Birt et al., 2017). Some authors also explicitly grounded their conceptual approach theoretically, most notably in the case of critical disability studies (e.g., Bartlett & O’Connor, 2007; 2010; Egdell et al., 2018), the application of the ethics of care framework to understanding citizenship in the context of dementia (e.g, Brannelly, 2011, 2016; Gilmour & Brannelly, 2010), and narrative theory (e.g., Baldwin, 2008; Baldwin & Greason, 2016).

Despite the different descriptors and theoretical underpinnings, thematic analysis reveals that there are core concepts recurring in all approaches. Five dominant themes emerged: 1) the relationality of citizenship; 2) the nuances of agency; 3) autonomy as facilitated; 4) focus on stigma, discrimination and exclusion; and 5) attention to the possibilities of identity and growth. Table 1 shows the data from the 49 articles that were extracted and categorized around these themes. The table also indicates whether papers are primarily conceptual or research-based. Upon further analysis, it was determined that discussions of agency and autonomy could be collapsed as they are closely connected. Hence, the following pages discuss the findings under four sub-headings: the relationality of citizenship; facilitated agency and autonomy; stigma, discrimination and exclusion; and identity and growth.

**Table 1.** Key Themes of Citizenship.

Author(s), year & Type of paper	The relationality of citizenship	Nuances of agency	Autonomy as facilitated	Focus of stigma, discrimination and exclusion	Identity and growth
1. Bartlett and O’Connor (2007)	Focus on examining lived experience of dementia and interpersonal relationships within the context of broader socio-political context	Focus on agency and power of the person living with dementia	Challenges previous positions (e.g., personhood) that frame people living with dementia as “passively dependent on others for affirmation” (p. 110)	Resisting injustice is a key aspect of citizenship in the context of dementia	Recognizes the need to focus on people’s multiple social roles and identities (and the power affiliated with different positions)
Conceptual paper	Introduces understanding of citizenship as social, not just political. Reframes citizenship as a social practice and constructed within one’s relationships with each other and society in general	Shift from traditional understanding of citizenship which excludes people living with dementia based on lack of agency to a view of people with dementia as people with power	More inclusive approach than traditional understandings of citizenship that excludes people based on capacity for self-determination	Focus on a) people’s right to be free of stigma and discrimination, and b) the facilitation of the right to freedom from discrimination through relationships and practice	Recognizes dementia as both a time of loss but also with potential for growth in other areas; Accounts for people changing and growing
2. Baldwin (2008)	Focus on: a)social relationships within the broader context of legal, political, and social rights, belonging, and contribution to society; b) interrelatedness of personal, interpersonal, and structural contexts, and c) people’s co-construction of self-narratives with others	Narrative agency: continued opportunity to tell one’s story from a subjective perspective, including the opportunity to form and control that narrative	Focus on: the recognition of narrative interdependency; and, supporting people with dementia to have the opportunity, time, and resources to develop their own narratives and become involved in policy- and decision-making processes	Highlights marginalization of the narratives of people living with dementia and enabling people with dementia to shape their own narratives	Focus on how master/policy narratives constitute an identity that is not aligned to the identities of people living with dementia (which in turn limits opportunities for participation and fulfilment)
Conceptual paper		Source of narrative agency includes alternative forms of expression, e.g., non-verbal expression			
3. Boyle (2008)	Takes into account broader relationships such as the legislative, policy-level, and social influences on people’s citizenship	Legislation can empower people with dementia when they retain capacity and protect them when they do not – but in restricted ways only	*Assisted autonomy:* Focus on provision of support in decision-making to enhance people’s exercise of autonomy and supporting both verbal and non-verbal communication of people living with dementia to maximize their decision-making capacity	Challenges the neglect and ill-treatment of people living with dementia	*Not explicitly covered*
Conceptual paper	Highlights need for care providers to receive guidance and training to conduct capacity assessment and support decision-making			Focus on ensuring (through the appointment of capacity advocates) that people living with dementia are not discriminated against on the basis of lack of capacity	
4. Behuniak (2010)	Focus on a) how different institutions exert power over individuals, and b) people’s actual power	Vulnerability framed not as a state of lack of power but as a state that warrants special care and protection due to lowered capacity, without loss of citizenship and rights	Recognizes both active and passive citizenship	Concerned with striking a balance between rights and protection in the context of decreasing capacity.	
Conceptual paper	Power is realized through interdependence with others.		Prioritizes supported decision-making rather than proxy decision-making, to ensure the person is involved	Emphasis on *vulnerable person* status (rather than ‘citizen’) to better account for individuals’ legal and ethical entitlements	*Not explicitly covered*
5. Boyle (2010) Conceptual paper	Calls for greater commitment at the policy level to maximize people’s ability to exercise self-determination and facilitating their rights in the context of care	Challenges common assumptions of absence of capacity, and equating dementia to lack of capacity Facilitating decision-making *by* people with lowered capacities themselves is necessary to promote autonomy	Focus on protecting people’s autonomy and right to self-determination in case of lower decision-making capacity	Challenges a)the denial of opportunities for self-determination based on inaccurate judgements of capacity, and b) the inappropriate exclusion of people living with dementia from decision-making (related to their care)	*Not explicitly covered*
6. Gilmour and Brannelly (2010)	Focus on power relations that shape the lived experience of dementia and seeks to politicize personal experience	Challenges the medical model’s positioning of people living with dementia as voiceless and instead privileges their agency and rights	Focus on relational autonomy : People living with dementia do not always make decisions about their lives individually, rather they are situated in a social network while making decisions	Focus on challenging stigma and discrimination	*Not explicitly covered*
Conceptual paper	Expands the notion of citizenship beyond political participation to also encompass social participation	Positions people as “experiencing speaking subject with desires, needs and agency” and as “active agents” (p. 245)	Frames care relationships through the lens of interdependence and negotiation and rejects notions of independence or dependence	In case people have needs and require care/support their position as citizens should not be immediately delegitimized	
7. Brannelly (2011) Research study	Societal discrimination results in the ‘social death’ of people living with dementia; A value-based approach by others challenges this and promotes participation of those living with dementia in society	A more traditional approach to citizenship is based on personal competence, characterizing people living with dementia as ‘passive actors’ and denying them the “possibility of active citizenship” due to the lack of engagement owing to lower cognition and communication skills (p. 663).	Emphasis on facilitating decisional autonomy Making informed proxy decisions for those who cannot adequately represent themselves is contingent on care providers’ knowledge of the person’s biographical narrative	Personal or cultural stigma surrounding dementia could also influence care providers’ behaviour with people Exclusionary practices signify social disregard and result in disempowerment.	*Not explicitly covered*
8. Orulv (2012)	Focus on solidarity and empowerment fostered through mutual support among people living with dementia	Focus on people living with dementia as agents of change	People living with dementia wish to be involved in and influence decision-making for as long as they are able to make informed decisions and choices and shape their own narratives	Focus on eliminating stigmatizing attitudes or practices	*Not explicitly covered*
Research study	Citizenship is facilitated by creating spaces for mutuality for people living with dementia to take the lead.	Power imbalances in interdependent relationships result in vulnerability and greater susceptibility for marginalization			
9. Kelly and Innes (2013)	Focus on the asymmetric power dynamics in relationships that limit people’s ability to exercise citizenship	Challenges the notion of citizenship based on productivity and ability to work, which positions people living with dementia as second-class citizens	Rights-based approach to dementia care focuses on facilitating autonomy and self-determination Focus on recognizing and challenging barriers to individual choice and control in care practice	Identifies macro-level constraints that deter providers from adopting a rights-based approach and impede the realization of human rights through care practices	Frames abuse, neglect, and discrimination as violation of human rights, dignity, and identity
Conceptual paper			Focus on recognition of and challenging barriers to participation		
10. Bartlett (2014a)	Focus is on people’s social positions and networks and leveraging them to influence decision-making and achieve personal goals	Focus on more active participation while acknowledging the exclusion of people who are capable of less-active forms of participation	*Not explicitly covered*	Experiences of stigma and discrimination can motivate people living with dementia to take action (e.g., involvement in campaigns)	Focus on a shared, distinctive, and collective identity formed through participation and engagement, and b) a sense of solidarity
Research study	Forging interpersonal connections to produce macro-level impact				Group identity used to further people’s ability to influence decision-making
11. Bartlett (2014b)	Focus on collective experience and others’ recognition of people’s right to participate is central to an inclusive approach	Citizenship should not be limited to being politically active and should be expanded to include people who are involved in more diverse ways	*Not explicitly covered*	Stigmatizing/discriminatory acts (e.g., invalidation of people’s diagnosis because they do not conform to stereotypes) is a barrier to citizenship practice	Emphasis on collective identity rather than self-identity; Solidarity as empowering and grounding
Research study		Need for more inclusivity to counter (biomedical) views based on deficit that limit participation		Focus on treating people living with dementia with equality and fairness and not as second-class citizens	
12. Nedlund and Nordh, (2015)	Examines how policy constructions of dementia can legitimize or challenge social constructions of citizenship of people living with dementia	Calls for a shift within policy narratives from a medical deficit-focused approach to one that is more inclusive and democratic	Challenges policy narratives that negatively frame dependence as a failure in terms of burden or cost	The lack of visibility and exclusion of people living with dementia in policy narratives limits the opportunities for people to exercise their citizenship, be valued and recognized, and influence decision-making	*Not explicitly covered*
Research study	Perceptions, attitudes, and assumptions regarding people living with dementia play a key role in framing them as citizens	Suggests framing people living with dementia (in the context of policy narratives) as capable citizens” to highlight their agency and power	To adopt a more dynamic citizenship approach that takes into account the progressively evolving nature of people’s cognitive capacities due to dementia		
13. Baldwin and Greason (2016)	Framing citizenship as a practice focuses on the relationships between people living with dementia and others.	Citizenship involves a claim not only to tangible rights to services and resources but also to agency, participation, and power	Involvement in individual or collective decisions (e.g., as part of steering committees, organizational governance, and decision-making forums) and having choice are critical to realize citizenship	Traditional understanding of citizenship (as a status) is based on inclusion/exclusion criteria	Citizenship practices help support a sense of identity and belonging
Research study	Constitutes actions and practices of individuals and collectives of people living with dementia at national, communal, and organizational levels	Acknowledges varied forms/levels of participation and not just being involved in an active manner	Draws in notion of interdependence: shift in seeing people as capable of not only receiving care but making meaningful contributions	Citizenship as a practice is a more inclusive approach as it does not involve inclusion or exclusion criteria	
14. Bartlett (2016)	Citizenship is shaped by relational aspects (inequalities) emerging both in the home as well as outside.	Emphasis on full participation of people living with dementia	Focus on the role played by each person in shaping citizenship, instead of viewing it solely as a status conferred upon people by the state.	Focus on struggle against injustice for fairness and equality. Micro-injustices within domestic spaces also play a part in shaping political citizenship at broader levels	Focus on opportunities for growth
Conceptual paper	.	Focus on ordinariness of citizen participation, going beyond political participation, and opportunities for growth		Suggests that stigma is the most prominent barrier to realizing citizenship in the community	
15. Brannelly (2016)	Citizenship of people living with dementia is relational to others	Integrity of care/guidance for practice is needed in order to “avoid domination” (p. 310) for people with dementia	Criticizes the denial of citizenship of people living with dementia on the basis of notions of reduced independence or autonomy.	Challenges societal norms that contribute to marginalization of people living with dementia	Citizenship is linked to people’s identity and maintaining/fulfilling their social roles that help render their lives as meaningful
Conceptual paper	Under-resourced social care has negative implications on the citizenship of older people living with dementia Draws in ‘ethics of care’ lens to position citizenship as relational and interdependent		Focus on promoting autonomy through interdependence. Shared decision-making challenges the notion of inability of people living with dementia	Focus on elimination of sources of oppression that undermine rights and citizenship and recognition of vulnerability to better understand inequalities and delineate additional rights for people living with dementia.	The maintenance of social roles can be challenging and results in exclusion
16. Russell (2016)	Findings from this study support a biopsychosocial context for understanding the lived experience of dementia via co-produced learning	People living with dementia acting as educators about the dementia experience by co-producing learning.	Participating in a learning initiative provided opportunities to enhance confidence	Involving people with dementia in post-secondary education can promote social justice, challenge discrimination, recognize diversity and increase inclusion	*Not explicitly covered*
Research study		Opportunity for them to engage as active social agents/address power imbalances			
17. Ward et al. (2016) Research study	Examines relationships that older women with dementia have with one another and with their hairdressers with a focus on embodied citizenship	Discusses creative and collective forms of agency, in particular control over appearance/appearance-related practices	The hairdressing salon enabled social participation for older women with dementia	Intersecting forms of resistance unfold in the hair salon – the everyday spaces in which citizenship is expressed and discrimination is resisted.	Paying attention to one’s personal appearance is integral to one’s identity/biography; Focuses explicitly on gender
18. Dupuis at al. (2016)	Citizenship is fostered through narrative and arts-based connections	Becoming aware and feeling empowered by continued abilities and possibilities, rather than focusing on “tragedy discourse” common with dementia	Demonstrates how arts-based and narrative activities can facilitate people to exercise citizenship	There is a need to challenge the tragedy discourse in dementia which perpetuates stigma and discrimination	Participatory arts can nurture personal growth
Research study	A relational process of opening up spaces for voices and stories, constructing alternative narratives of dementia through the arts			Activist efforts in challenging entrenched discourses contributes to the sense of being a citizen in the community	Social contexts can foster the co-construction of new narratives
19. Clarke and Bailey (2016)	Citizenship is constructed through people’s everyday social practices and interactions with family members, wider community, and physical environment	Familiarity with the physical and social environment (history of living in the same area) both negatively and positively influences active citizenship	Focus on preservation of autonomy as an integral part of citizenship and challenges the approach of focusing on individual-level deficits and incapacity by adopting a more relational approach	Stigma is a barrier to maintaining a sense of normality and belonging to the community. It hinders social engagement, which is integral to citizenship practices	Focus on preservation of identity as an integral part of citizenship
Research study		A reciprocal approach focuses not only on the supports that people require but also their strengths and assets (p. 435)	Ability to adapt and make decisions that protect one’s self-esteem and dignity may be limited or not fostered by others	Others’ stigmatizing or exclusionary attitudes and behaviour diminishes agency of people living with dementia	Citizenship is affected by personal histories and changes in people’s social roles, identities, and their sense of belonging to the community
20. Kontos et al. (2016)	Focus on the role of embodiment in reciprocal engagement	Focus on self-expression through embodiment as more inclusive; and embodied agency, or self-hood where decision-making ability is not predicated upon cognitive capacity but also stems from pre-reflective aspects of human condition.	Questions that autonomy should be based on the ideal of self-determination and people’s ability to make informed choices on their own	Challenges the denial of citizenship rights of people living with dementia	*Not explicitly covered*
Conceptual paper	Requires others to support people’s right to self-expression while protecting them from harm.		Suggests this understanding results in people in later stages of dementia being excluded	Embodied self-expression is fundamental to the human condition and must be supported as a human right	
21. Nedlund and Larsson (2016)	Draws attention to the mutuality between being an independent person with rights in society *and* receiving support from the welfare system.	Highlights the exclusionary nature of a citizenship approach that assumes capacity for self-determination.as a pre-requisite	Highlights the difficulty of ascertaining whether someone has the capacity for self-determination or has decision-making incapacity and needs assistance.	Predicating participation upon intact capacity for self-determination results in inequality and exclusion	*Not explicitly covered*
Conceptual paper	Highlights legal constructions of citizenship and how it informs decision-making and maintaining best interests of people living with dementia	Challenges the norm of citizenship having to be active and the assumption that citizens have the capacity to represent themselves.	Advocates for a supportive decision-making approach as a means for enhancing citizenship across all stages of the dementia experience		
22. Österholm and Hydén (2016)	Citizenship is expressed through people’s communication with care providers	Focus on people’s ability to communicate their needs and cope with communication difficulties	Health and social care providers assumptions regarding people’s capacity are integral to the facilitation of autonomy and choice in decisions.	Citizenship of people can be taken from them when there are communication problems	*Not explicitly covered*
Research study		Recognition that different people have different styles of communication and show different levels of participation	Care providers can play a supportive role in facilitating the communication of people living with dementia.		
23. Phinney et al. (2016) Research study	Participation of people living with dementia framed as “claiming their rightful place in the broader community” (p. 390)	Simultaneous focus on recognizing people’s vulnerability and diminished capacity, as well as their dignity and respect	Emphasis on the importance of finding ways to prioritize both safety and free/autonomous participation of people living with dementia	Focus on resistance of labelling persons as having a dementia diagnosis. Programs based on activities that anyone, not just people living with dementia, would enjoy helps reduce stigma	Focus on shared social identity
24. Sonnicksen (2016)	Focus on representation: making decisions on behalf of others in their best interests	Challenges the notion of cognitive capacity as a prerequisite for includability in democratic participation	Challenges “ableist biases on autonomy and decision-making capacity” (p. 331)	Exclusion of people with dementia violates the principles of democratic citizenship, including equality, representation, participation, and inclusion	*Not explicitly covered*
Conceptual paper	Political approach to conceptualizing citizenship	The participation of people living with dementia in a democracy depends on the support of others, especially in the later stages of dementia	*Group-based collective self-determination* calls for policy and decision-making to be influenced by people living with dementia and care partners		
25. Wiersma et al. (2016)	Addresses how relationships with family carers may foster or impede one’s sense of self as a full citizen when living with dementia.	Self-management groups for people with dementia provide spaces that help maintain people’s voices and position them as actively participating in their own lives.	People living with dementia should play a key role in decision-making Flexibility and choice are key in supporting citizenship practice	Emphasis on ensuring that people living with dementia do not feel marginalized and that their voices are heard	Recognized that ‘one size does not fit all’. Approaches to facilitating citizenship must consider the person living with dementia as an individual
Research study	The relationship dynamic between people living with dementia and their care partner could influence their ability to exercise citizenship	Repositioning people living with dementia as individuals with experiential expertise helps challenge the notion of people as passive actors and recast them as learners, knowers, and teachers	In group work, some separation between those living with dementia and care partners may be important to create a safe and empowering space of solidarity and belonging for people living with dementia	Peer support helps frame stigma as a shared experience, suggesting that people living with dementia are not alone in their experiences	
26. Birt et al. (2017)	Shift from focus on individual experience to the socio-political forces	Social structures can facilitate or inhibit people’s agency	Challenges deficit-focused narratives that strip people of their autonomy and exclude them from decision-making.	Challenges the exclusion of people living with dementia from participation in social practices	Social roles and statuses change as people move along the dementia trajectory
Conceptual paper	Participation of people living with dementia should be recognized and acknowledged through the action of others	Citizenship must consider both the active and passive forms of agency that people with severe dementia may display	Focus on support in enabling “purposeful contribution to decisions” (p. 206)	How cultures frame dementia could result in challenges to people’s autonomy and lead to stigma and exclusion	Focus on people’s management of disclosure of dementia diagnosis to facilitate control in social situations and continuity of social roles
27. Grenier et al. (2017)	Focus on sociocultural assumptions and structural inequalities as forces that shape citizenship	Challenges previous deficit-focused positioning of people in the later stages of dementia as lacking agency or potential, which furthers the marginalization of people.	This approach challenges the ideal of independence and offers a more inclusive model grounded in vulnerability to better represent the lived experience of people in the late stages of dementia	Calls for a paradigm shift in the provision of care and services that is based not on pity but on valuing people living with dementia	*Not explicitly covered*
Conceptual paper	Contextualizes life decisions in the context of power relations and social values, institutions, and practices. Emphasis on others treating people who are vulnerable with care and compassion	Recognition of vulnerability is framed as a shared responsibility and is key in the effort to maintain agency and dignity	Challenges negative valuation of dependence, which tends to devalue and other people living with dementia in the fourth age		
28. Kelson et al. (2017) Research study	Examines the role of public art in facilitating social citizenship and sense of community belonging for people with dementia	Social participation and inclusion foster health, wellbeing and quality of life for people with dementia – and art in public spaces can create opportunities for people with dementia to participate in the community at large	*Not explicitly covered*	Inclusive communities respect people with dementia as social citizens with rights to participation and contribution	*Not explicitly covered*
29. Kontos et al.(2017) Research study	Relationship-centred care a) includes attention to interdependence, reciprocity, and involvement of people with dementia in decision-making; b) power relations between people living with dementia and others, and, c) interpersonal influences at the micro-level, as well as macro-level factors that impinge on people’s self-expression	Embodied agency: the inherent capacity of the body as the primary source of agency; Participation is promoted by supporting and nurturing people’s embodied capacity	Shift from autonomy and individuality to interdependence and relationality. Offers a relational perspective to autonomy	Stigma and discrimination pose barriers to people’s self-expression Citizenship approach is key to eliminating discriminatory practices in long-term care settings	*Not explicitly covered*
30. Bartlet et al. (2018)	Considers gender differences among people living with dementia in the context of care in order to promote equality (in terms of access to resources, decision-making, roles and responsibilities)	Focus on gender differences of agency and the restrictions placed on social participation	Focus on gender differences in facilitation of autonomy by care partner	Focus on social exclusion, disablism, and inequality through the lens of gender	Addresses gender
Scoping review	Focus on the influence of social position on the lived experience of dementia			A feminist citizenship perspective in future research could provide a framework for working towards justice and equality	
31. Egdell et al. (2018)	Focus on recognition of dementia as disability (by expanding the definition of disability) in legal frameworks to help achieve equity and justice for people	Supporting people’s exercise of legal capacity is integral to inclusion and participation.	Supporting people’s exercise of legal capacity is integral to the maintenance of autonomy	Focus on disability discrimination and making reasonable adjustments to practices that place people living with dementia at a disadvantage and hinder their full and effective participation in society on an equal basis with others	*Not explicitly covered*
Conceptual paper		Challenges negative perceptions of people’s competence and capacity			
32. Grigorovich and Kontos (2018)	Focus on relationality and reciprocity	Challenges the assumption of loss of agency due to dementia	Focus on interdependence in decision-making	Focus on elimination of stigma and discriminatory attitudes and practices in the care context	*Not explicitly covered*
Conceptual paper	Frames people living with dementia as active partners in care relationships.	The body is framed as a source of capacity for intentional and meaningful action			
33. Ursin and Lotherington (2018)	Conceptualizes relational citizenship as distributed achievement from a network of relations (care-collectives) rather than viewing the individual as holding citizenship	Distribution of agency occurs through care-collectives – and this can be empowering or harmful	*Not explicitly covered*	*Not explicitly covered*	Focus is on care-collectives rather than on the individual
Research study		Regardless, it is a shared or collective achievement			
34. Kelly and Yarwood (2018)	Shift from viewing citizenship solely in the context of political participation to that of engaging in everyday social practices	Frames citizenship as people’s ability to engage in social practices	*Not explicitly covered*	Aspects of the lived experience of dementia are socially constructed and may result in exclusion	Citizenship is more strongly associated with personal identity than people’s sense of belonging to the community
Research study	Citizenship practices are located at the intersections of different (micro and meso) levels of the environment				Sense of belonging may diminish with changes in the community causing people to feel out of place
35. Kontos and Grigorovich, (2018a)	Embodied selfhood is linked to people’s social world and is, therefore, relational	Challenges the assumption of loss of agency by suggesting that the “creative and intentional capacity” (p. 718) remains preserved in the body and is not lost due to cognitive impairment	Focus on corporeality as integral to self-expression	People’s behaviour and actions could be indicators of stigmatizing care practices	Creative activities and arts-based activities in dementia care, such as dance, can facilitate expression of self for people living with dementia
Conceptual paper	A relational lens helps focus on reciprocal engagement		Relational lens helps focus on interdependence	Focus on counteracting assumptions about people’s capacity	Adopting this approach would facilitate “creativity, imagination, and other positive human potentialities” of people living with dementia (p. 41)
36. Kontos and Grigorovich (2018b)	Embodiment is a source of reciprocal engagement	Embodiment is a source of self-expression	Embodiment is a source of interdependence		
Research study	Embodied selfhood is relational and “intertwined with [the] shared world” (p. 41)	Argues against assumptions of loss of agency in dementia by demonstrating the persistence of agency through the pre-reflective capacity of the body			
37. Marsh et al. (2018)	Power imbalances are levelled by volunteers at a gardening program forming reciprocal relationships with people living with dementia “to collectively look out for each other, and keep people safe by getting to know and understand each other” (p. 178)	Assuming that the person has capacity rather than assuming that they are not capable, that they are able, rather than disabled.	People should have the opportunity to exercise autonomy and choice in negotiating risk	Challenges the approach of creating dementia-only programs/spaces as these further the divide between people living with dementia and the community.	*Not explicitly covered*
Research study		Autonomy requires acknowledgement of people’s strengths and contributions	Focus on facilitating people’s autonomy and their right to freely choose to take risks; ccommunities should enable risk-taking	Instead, calls for full inclusion including spaces/services that can be used by people living with dementia and others.	
38. O’Connor et al. (2018)	Citizenship is shaped through one’s everyday interactions with one’s world. Societal attitudes shape interpersonal and personal responses to dementia in meaningful – and often negative – ways.	Focus on the right of the person living with dementia to be treated as an agent with “rights, history, and competencies” (p. 46)	Disclosure of one’s diagnosis of dementia can result in one being immediately treated as incapable but there is also power in strategically owning one’s diagnosis as a means of claiming citizenship.	Disclosing one’s diagnosis of dementia can result in discrimination by others. Strategically initiating dialogue with others about their diagnosis, people play the role of educating others on how to be supportive and challenge stigma.	People play an active role in shaping their social identity (by disclosing their diagnosis). Internalized oppression – people may unintentionally take up how society treats them so that they begin to feel less capable.
Research study		Others’ perceptions of dementia may limit people’s opportunities for active participation and challenge their citizenship.			Others’ perception and assumptions about dementia may serve to limit the growth of people living with dementia.
39. Grigorovich et al. (2019)	Focus on the role of embodiment in relationality and reciprocity	Focus on (a) supporting relational and embodied capabilities of people living with dementia, and (b) embodied self-expression and pre-reflective intentionality and agency of people living with dementia	Focus on the role of embodiment in interdependence	Challenges stigmatizing perceptions of dementia (e.g., highlighting dementia and the individual as the cause of aggression)	Focus on human flourishing as a goal of dementia care.
Conceptual paper	Situates interpersonal relationships and interactions in the care setting within wider social, organizational, political, and institutional context		Going beyond basic physical safety and comfort in care and instead focusing on the best interests of people living with dementia		Focus not only on protection from harm but also on creating a relational care environment that supports the positive potentialities (e.g., creativity, imagination) of people living with dementia
40. Keyes et al. (2019)	Citizenship is realized through social participation through everyday interpersonal interactions	Focus in on deliberative/creative agency	The freedom to make decisions is central to citizenship. Changes in decision-making abilities do not imply an end to people’s involvement in decision-making (e.g., supported/substituted decision-making)	Dementia is framed as “an experience within which people face barriers [rooted in institutional and social practices] to inclusion which impact upon their day-to-day lives” (p. 297)	Interdependent relationships help reinforce the identity of people living with dementia, which in turn, contributes to citizenship
Research study	Relational citizenship challenges the notion of independence and the dichotomous understandings of dependence and independence		Relational citizenship focuses on maintaining interdependence and reciprocity in interpersonal relationships within domestic settings	Challenges: (a) discriminatory practices that limit people’s participation; (b) conflating diagnosis with incapacity without proper/holistic assessment	
41. O’Connor (2019)	Frames mental capacity as a relational concept	Argues for presumption of capacity as a legal right; Recognizes capability as complex, not an ‘all or nothing’ and situational	Decision-making capacity should not be decided solely on the basis of cognition but should also take into account the person’s interactions with others and their environment.		Recognizes the challenge of maintaining the balance between acknowledging continuity post-diagnosis and the possibility of growth and change of mind when considering decision-making
Conceptual paper	Personal beliefs, values, and preferences are shaped by broader sociocultural contexts. Therefore, their influence on people’s decision-making need to be understood relationally	Advocates for an understanding of autonomy as relational	Disputes the dichotomy between dependence and independence and focuses on interdependence.		Recognizes that people living with dementia occupy different social locations and are not a homogenous group. These social locations help explicate why people respond to certain experiences a certain way
	People have rights even when incapable of making some decisions		Recognizes the importance of active involvement in decisions about one’s life even if not legally fully capable of making those decisions		
42. Seetharaman and Chaudhury (2020)	Partnerships with decision and policy makers needs to be authentic for advocacy to be meaningful for advocates living with dementia	Advocacy can only occur when people with dementia recognize their ability to be active citizens	Advocates living with dementia need to be meaningfully involved in decision-making	Study uses a human rights perspective to explore how people with dementia become advocates	Supporting engagement and advocacy for people with dementia acknowledges their identity as social citizens
Research study		Support from others (ie: organizations) is also required for opportunities for advocacy to occur	Others (planners, community partners, etc…) need to appreciate and value the contributions of people with dementia in these processes		Personal growth (building new skills such as advocacy) is an important aspect of citizenship
43. Kontos et al. (2020)	Draws on relational model of citizenship whereby embodiment becomes the course of relationality and self-expression	Focuses on embodied intentionality and how the body creatively engages with the world.	Arts-based programming is important for facilitation creative of -expression for people with dementia	Embodied self-expression for people with advanced dementia becomes primary way of engaging with world – this is important for supporting human rights (freedom of expression and human dignity)	*Not explicitly covered*
Research study		The body as a source of agency	Creativity should be nurtured in all aspect of life (need for enabling environments)		
44. Mitchel et al. (2020) Conceptual paper	Relational practices are needed in dementia and long-term care that honour full citizenship of people with dementia Relational citizenship as alternate care ethic	Agency is separate from cognition – can be found in the pre-reflective body	Authors argue for a replacement of more traditional views of autonomy with one of relational autonomy (interdependence and reciprocity	Relational practices create “a more human world for persons living with memory loss” (p.1) and challenges tragedy and biomedical discourse	Stigma has contributed to “spoiled identity”
45. Robertson and McCall (2020) Research study	Draws on relational citizenship model for embodied learning and creativity	Reciprocity and interdependence promote agency	People with dementia should be active partners in their care supports	There is a need for affirming creative self-expression and having the opportunity to continue to learn and grow	Arts-based activities can affirm and reinforce identity
46. Ursin (2020) Research study	Citizenship occurs through ‘care collectives’ of various care relationships that are heterogenous and changeable networks made up of different actors	Agency as relational care practices - “a collective and distributed construction emerging from the interplay of care collectives” (p. 3)	People with dementia should be co-creators of their everyday lives – this can be facilitated within care work	Care and care work need to shift to ensure that people with dementia feel included and able to participate	*Not explicitly covered*
47. Mann (2020) Conceptual paper	Not explicitly covered	People with dementia have the right to give own consent as long as they are able to	Charter of rights challenges common assumptions of incompetence and incapability	Growing recognition of the need for a human rights approach for people with dementia	*Not explicitly covered*
48. Mitra and Schicktanz (2020) Research study	Looked at micro-citizenship interactions of patient support organizations (ie: dementia cafes, support groups, online groups, etc….)	*Not explicitly covered*	*Not explicitly covered*	Support organizations can support people with dementia to claim rights but the extent of that support depends on funding and resources	Patient support organizations have a role in helping people construct “citizenship identities” (that recognize age, gender, ethnicity, etc…)
49. Bartlett (2021) Research study	Focus in relationship to wider societal and organizational structures	Agency can be distributed among humans and non-humans (ie: animals and technology)	Citizenship involves the “conscious sharing of responsibilities between people, technological beings, even animals…” (p12)	Access (to resources, places and opportunities) is a key element in inclusive citizenship	There is value in acknowledging a “disabled identity”

### The relationality of citizenship

A core foundation of all reviewed literature is the importance of relationships and how they can position individual and collective experiences, rights, and responsibilities. In fact, relationality is an overarching theme that connects all of the other themes and involves recognizing the varying, complex and synergistic relationships that contribute to citizenship. This is not unexpected – discussions of citizenship pick up on the relationally-oriented personhood literature. What emerges as different in the citizenship literature though is the move beyond interpersonal relationships to consider broader institutional and societal responses.

In many instances, relationality is framed within the social citizenship perspective as first developed by Bartlett and O’Connor (2007; 2010). Prevalent in this perspective is citizenship as *social practice*, specifically the relational and dynamic practice through which people living with dementia relate to fellow citizens, and are connected to the community and society-at-large (Bartlett & O’Connor, 2007, 2010; Brannelly, 2011; Phinney et al., 2016). Importantly, this can include everyday social practices and interactions in the community (e.g., home, neighbourhood, care settings), thus shifting more traditional citizenship perspectives situated in civil and political participation to a wider range of practices of cohabitating and interacting with others (see for example: Baldwin & Greason, 2016; Bartlett, 2016, 2021; Birt et al., 2017; Brannelly, 2011; Clarke & Bailey, 2016; Gilmour & Brannelly, 2010; Kelly & Yarwood, 2018; Keyes et al., 2019; O’Connor et al., 2018; Seetharaman & Chaudhury, 2020).

The concept of relationality in citizenship was further extended by Kontos and colleagues in their model of relational citizenship (Kontos et al., 2016). Drawing in earlier work revolving around embodied selfhood (Kontos, 2005), relationality is intertwined with recognising how people with dementia express their sense of selves via their bodies (Kontos et al., 2016). This intra-relational perspective has been important in extending the citizenship discourse for people living with advanced dementia, for whom embodied self-expression and the relationality of human connection become critical means of engagement as citizens in the world (Grigorovich et al., 2019). This challenges dominant narratives that premise creativity purely on individual cognition, and instead, foreground the interaction between pre-cognitive and pre-reflective capacities of the body and an enabling environment as being central to the everyday creativity of people living with dementia (Kontos et al., 2020). The embodied citizenship lens has been used to explore creative, artistic and sexual expression of citizenship for people living with dementia (Grigorovich & Kontos, 2018; Kontos et al., 2017; Kontos & Grigorovich, 2018a, 2018b).

Whether envisioned or understood as a social practice or embodied experience, there is emphasis in this body of literature on citizenship as relationally enacted in different spaces and places. For example, Kelly and Yarwood (2018) explore what it means to be a citizen living with dementia in rural environments, focusing on how citizenship unfolds in rural contexts and in the way “people engage with society, its social practices and daily routines” (p. 103), while Kelson et al. (2017) discuss practices of social citizenship in the context of public art in an urban setting and Ward et al. (2016) consider expression of citizenship and the relationships that older women with dementia have with one another and with their hairdressers in hair salons. Bartlett (2016) calls for a consideration of citizenship “within the practice of ‘ordinary’” (p. 455), focusing on citizenship within domestic contexts and the micro injustices that can occur in these daily and relational spaces which can erode citizenship. Clarke and Bailey (2016) build on this by explicating how people’s long-term knowledge of and familiarity with the physical-social environment of the community helps facilitate their citizenship practices, while changes in the environment over time could cause feelings of estrangement, which in turn, undermine citizenship. In addition to this focus on day-to-day space, a smaller body of research has explored how decision making and considerations of capacity for people with dementia occur within legal and political spaces and can be understood relationally (for example, Boyle, 2010; Nedlund & Larson, 2016; Sonnicksen, 2016; O’Connor, 2019). Recognizing the importance between relationality and temporality, the importance of safe spaces, such as advisory or mutual aid groups of people living with dementia, as places for preserving citizenship by ensuring individuals feel comfortable and safe expressing ideas, thoughts and feelings, has been examined (for example, Örulv, 2012; Wiersma et al., 2016). This includes the concept of care-collectives, conceived as a network of “certain agents brought together in time and space, shifting along with changes in the relations comprising them” (Ursin & Lotherington, 2018, p. 63), further expanding understandings of the relational processes involved in citizenship, and the various ways in which they can occur. In the sections that follow, these ideas related to relationality will be further developed.

### Facilitated agency and autonomy

A second, interrelated theme is focused on reconceptualizing the closely related concepts of agency and autonomy in relation to citizenship of people living with dementia. Whilst agency generally referenced taking action (process) and autonomy was linked to independence (status), it was often unclear in this review that people were using the language of agency and autonomy in differentiated ways. Hence, for practical purposes, we have collapsed the two terms into one theme.

Overall, within this body of literature, there is emphasis on the adoption of an “unconditional and inclusive” approach to the conceptualization of agency (Bartlett & O’Connor, 2007, p. 114; see also,; Bartlett, 2014a; Grigorovich et al., 2019). Accordingly, people living with dementia are repositioned as capable of communication, participation, and contribution on some level (Baldwin & Greason, 2016; Marsh et al., 2018; Nedlund & Nordh, 2015; Österholm & Hydén, 2016), and also capable of desires, needs, power, competencies, experiential expertise, and rights to challenge the status quo and effect change (Bartlett & O’Connor, 2007, 2010; Gilmour & Brannelly, 2010; Nedlund & Nordh, 2015; Örulv, 2012; O’Connor et al., 2018; Wiersma et al., 2016). This literature consistently rejects the notion that the agency of people living with dementia be framed through a deficit-focused medical lens focusing solely on cognition. Such a view is perceived as exclusionary and incomplete, restricting the right to participate in one’s own life, resulting in denial of citizenship (see for example, Bartlett & O’Connor, 2007, 2010; Brannelly, 2011; Nedlund & Larsson, 2016). Overall, the literature specifically challenges the cognitive biases that position people with dementia as incapable and lacking awareness, subjectivity and decision-making abilities (Baldwin, 2008; Boyle, 2008; Gilmour & Brannelly, 2010; Grigorovich et al., 2019; Grigorovich & Kontos, 2018; Kontos et al., 2016; Sonnicksen, 2016). Many of the articles challenge the notion that citizenship is simply a status conferred upon people, and instead, emphasize people’s active involvement in making decisions and taking action, drawing attention to citizenship as a process. For example, the idea of “active citizenship” is generally understood as a right to full participation in everyday life and in activities in the wider socio-political arena. Active forms of participation may be expressed in the case of people living with dementia, for example, “tak[ing] control of social situations” and “actively manag[ing]” the decision to disclose their diagnosis (Birt et al., 2017, pp. 202–203; see also; O’Connor et al., 2018).

Implicitly, and in some cases explicitly (for example, Bartlett & O’Connor, 2007), the person living with dementia in this body of literature is positioned as more than just a passive ‘recipient of care’. However a precarious balancing act in considering agency and autonomy within the context of dementia emerges: Specifically, supporting agency and autonomy in dementia is identified as an ongoing process of preserving and maintaining people’s abilities and capacity for action for as long as possible while also recognizing the inevitability of changing/evolving abilities due to dementia progression (Bartlett & O’Connor, 2007, 2010; Keyes et al., 2019; Nedlund & Nordh, 2015; O’Connor, 2020). Recognizing this complexity, this body of literature has grappled with the need to conceptualize participation across a continuum, drawing attention to the ‘less active’, ‘nuanced,’ or ‘passive’ forms of participation of those who are further along in their dementia trajectory. For example, notions of narrativity and embodiment have been invoked to draw attention to how people with dementia may enact subtle forms of agency and autonomy even in the later stages of dementia (see for example Baldwin, 2008; Baldwin & Greason, 2016; Clarke & Bailey, 2016; Dupuis et al., 2016; Grenier et al., 2017; Grigorovich & Kontos, 2018; Keyes et al., 2019; Kontos et al., 2020; Kontos & Grigorovich, 2018a; Robertson & McCall, 2020). Birt et al., (2017) note that the “passive mode” of citizenship shows a greater emphasis on facilitation by others, whereby “the actions of others recognize and acknowledge the nuanced ways in which people with severe dementia may display agency” (p. 205). Hence, the role of others in supporting agency and facilitating autonomy is particularly prominent in this body of literature, explicitly addressing how interpersonal, social, political, and legal structures can either facilitate or inhibit citizenship of people with dementia (Bartlett, 2014a, 2014b; Birt et al, 2017; Gilmour & Brannelly, 2010; Kontos et al., 2016, 2017; Ursin & Lotherington, 2018).

This begins to extend discussions related to agency and autonomy in important ways. Firstly, much of this literature explicitly challenges exclusionary approaches to citizenship that draw on dichotomous understandings of independence and dependence. Instead, agency and autonomy are reframed through the lens of *interdependence*. For example, Mitchell et al. (2020) argue for replacing more traditional views of autonomy with one that is relational, consisting of interdependence and reciprocity, and Brannelly (2016) draws on an Ethics of Care framework as a conceptual heuristic for conceptualizing this shift. This approach allows others to not only see people living with dementia as requiring support in making decisions, but also as being capable of making meaningful contributions to decision-making. For example, Baldwin (2008) proposes the notion of narrative interdependency, which involves supporting people living with dementia to have the opportunity, time, and resources to develop their own narratives and help shape policy narratives by becoming involved in decision-making processes. These ideas are being translated into new approaches to care, suggesting that adopting an interdependence-based approach in relationships of people living with dementia and their care partners helps facilitate people’s control and choice through collaborative decision-making (Brannelly, 2011; Keyes et al., 2019; O’Connor, 2020).

Secondly – and extending the notion of interdependence - this body of literature conceptualizes agency and autonomy through the interplay of rights, resilience, and protection, emphasizing the need for a balance between supporting people’s right to take risks and cope with challenges, and ensuring that they are safe and cared for when not able to do so themselves (see Baldwin & Greason, 2016; Behuniak, 2010; Brannelly, 2016; Clarke & Bailey, 2016; Marsh et al., 2018; O’Connor, 2020; Phinney et al., 2016). These authors emphasize the value in recognizing vulnerability and dependence across the dementia trajectory to better understand power relations and inequalities, and delineate additional rights that address the specific needs and challenges of people living with dementia. However, tensions emerge regarding how this balance is understood and addressed. Reconceptualizing care and protection as ʻpractices that impinge on human rights that people may object toʼ (Brannelly, 2016, p. 309) gives people living with dementia the opportunity to provide their consent to receiving care and have greater choice, control, and autonomy in care decisions (Nedlund & Larsson, 2016). O’Connor (2020) recognizes that while people living with dementia can be *deemed* incapable of making *some* decisions in their lives, they may still be involved as active participants in those decisions and quite capable of making other decisions.

It is suggested that recognizing vulnerability and protection would make for a more inclusive model of citizenship (see for example, Behuniak, 2010) and help maintain, recognize, and support the agency and autonomy of all people living with dementia (Grenier et al., 2017) but to date, there is only limited direction in this body of literature as to how to achieve this.

### Stigma, discrimination and exclusion

Perhaps one of the clearest defining features of the citizenship and dementia discourse is the emphasis on positioning stigma, discrimination and exclusion as a critical aspect of the dementia experience. All reviewed articles draw attention to these issues in some way, framing them as being in violation of people’s rights and the principles of citizenship. Specific aspects discussed related to this include: misuse of power (Bartlett & O’Connor, 2007); furthering negative stereotypes about dementia, for example, conflating diagnosis with incapacity (Bartlett, 2014a; Behuniak, 2010; Birt et al., 2017; Grenier et al., 2017; O’Connor et al., 2018); placing the onus of systemic failure (e.g., resident-to-resident aggression in long-term care) on the individual and dementia (Grigorovich et al., 2019); othering, devaluing, and de-legitimizing people’s position as citizens on the basis of vulnerability and dependence (Brannelly, 2011; Gilmour & Brannelly, 2010; Grenier et al., 2017); imposing restrictions on participation based on inaccurate judgement or assumption of capacity (Birt et al., 2017; Boyle, 2008, 2010; Nedlund & Larsson, 2016; O’Connor, 2020); and mis-representation of people living with dementia in policy narratives (Baldwin, 2008; Nedlund & Nordh, 2015). The importance of recognizing how social location (Bartlett & O’Connor, 2010; O’Connor, 2020), including gender inequities (Bartlett et al., 2018), may inform these experiences of stigma and discrimination in diverse ways is being recognized as an important area for further development.

Resisting and eliminating sources of injustice and oppression and facilitating people’s right to be free of stigma and discrimination are framed as central to the citizenship of people living with dementia (Bartlett & O’Connor, 2007, 2010; Brannelly, 2016). Some of the articles reviewed made recommendations to promote freedom from stigma, discrimination, and exclusion. These included: legal recognition of gross (more overt) violation of rights, as well as subtle (less obvious) forms of stigmatizing and discriminatory perceptions, attitudes, and practices in day-to-day practices (ie. Kelly & Innes, 2013); appointing third-party capacity advocates to ensure that people living with dementia are not discriminated against on the basis of lack of capacity and prevented from participation in decision-making (ie. Boyle, 2008); and ensuring that all assessments of decision-making capacity begin with the presumption of capacity (ie. O’Connor, 2019).

This body of literature draws attention to the possibilities that personal experiences of stigma and discrimination may motivate people living with dementia to take action through advocacy and education (see for example, Bartlett, 2014b; O’Connor et al., 2018; Russell, 2020; Seetharaman & Chaudhury, 2020). However, it also recognizes that stigma associated with the diagnosis and systemic barriers may pose challenges to people’s efforts to resist discrimination, thus necessitating adequate external support (Egdell et al., 2018; Kontos et al., 2017). The literature also suggests the empowering nature of peer interaction and support helps people recognize their shared experiences of stigma and discrimination as consistent with a citizenship lens (Örulv, 2012; Wiersma et al., 2016).

### Identity and growth

While the review found emphasis in the literature on continuity and maintenance of social people’s identities, multiple authors (Bartlett, 2016; Bartlett & O’Connor, 2007, 2010; Grigorovich et al., 2019; Kontos & Grigorovich, 2018a, 2018b) also focus on the opportunities for growth and development as an inherent right for those with dementia. Specifically, Bartlett and O’Connor (2010) challenge the notion of fixed sense of self that fails to account for the possibility of changes not only due to cognitive decline, but also in terms of shifting priorities or desires as the dementia progresses. In relation to the goals of dementia care, a minority of the articles reviewed explicitly argue against a narrow focus on ‘maintaining’ and call instead for the promotion of human flourishing and supporting positive potentialities such as creativity and imagination of people living with dementia (Grigorovich et al., 2019; Kontos & Grigorovich, 2018a, 2018b). O’Connor (2019) discusses the right of people living with dementia – similar to everyone else - to grow and change their mind.

Several articles (Baldwin & Greason, 2016; Bartlett, 2014b, 2014a; Bartlett & O’Connor, 2010; Birt et al., 2017; Brannelly, 2016; Clarke & Bailey, 2016; Kelly & Yarwood, 2018; Keyes et al., 2019; O’Connor et al., 2018) suggest that the continuity of identity, fulfilment of social roles, and maintenance of a sense of belonging and solidarity to the community affect people’s citizenship. According to these articles, citizenship is influenced by: “the interaction between engagement/participation, meaning-making, and identity, as experienced through one’s life story” (Baldwin & Greason, 2016, p. 293, see also; Bartlett & O’Connor, 2010) and, the tension between the maintenance of social roles and evolution of identities over the course of progression of dementia (Birt et al., 2017; Brannelly, 2016; Clarke & Bailey, 2016). Citing the discrepancy between the representation of people living with dementia in policy narratives, and the subjective narratives based on their lived experience and identities, Baldwin (2008) calls for bridging the gap between citizenship and self. Bartlett and O’Connor (2010) suggest that the values and meanings that the person living with dementia considers to be important shape their participation and actions as citizens, and therefore, emphasize the concept of sense of purpose as a central component of citizenship, which varies from person to person.

The articles reviewed suggest that a citizenship approach that focuses on identity helps reveal individual differences and disaggregates the category of people living with dementia, especially by drawing attention to the importance of social location (Bartlett & O’Connor, 2007). At the same time, it also facilitates collective and shared identities that foster a sense of solidarity (Bartlett, 2014a, 2014b; Wiersma et al., 2016). Bartlett and O’Connor (2010) extend the idea of taking account of the multiple identities of people living with dementia drawing on intersectionality theory to suggest recognize that the concept of social location as more representative of how people’s power relations with others shape the way they construct their identities and are perceived by others. The importance of moulding a citizenship approach that is more intersectional and sensitive to diversity and difference, for example, acknowledging how race, gender, socio-economic status and ethnicity (to name a few) help construct the experience of living with dementia in a particular way, is identified as grossly in need of further work (Bartlett et al., 2018; Bartlett & O’Connor, 2007, 2010).

## Discussion

All of the reviewed literature touches on aspects of Bartlett and O’Connor’s (2010) original definition of social citizenship, in terms of considerations of citizenship as a relationship or practice of some kind. Similarly, the articles reviewed in this paper stress the importance of upholding the rights of people living with dementia, as well as the need for individuals to be free from stigma and discrimination. There is also emphasis on citizenship being equated with opportunities for growth, as well as being able to participate in meaningful ways in life. Overall, this scoping review found a major emphasis on expanding definitions of agency and autonomy to render citizenship unconditional and fully inclusive of the diverse life experiences of people living with dementia. The alternative conceptualizations of agency and autonomy help introduce greater equity and fairness in citizenship practices and eliminate discrimination based on cognitively-biased standards.

The articles positioned people living with dementia differently: a) as active agents with power (Bartlett, 2014a, 2014b; Marsh et al., 2018; Wiersma et al., 2016); b) as people who are vulnerable (Behuniak, 2010; Brannelly, 2016; Grenier et al., 2017), and c) as both active and passive participants (Baldwin & Greason, 2016; Bartlett & O’Connor, 2007; Birt et al., 2017; O’Connor, 2019). This variation in positionality could be attributed to the emphasis on different stages of the dementia experience. For example, most articles focused on citizenship in the early stages, whilst fewer focused on the late stages of dementia. One particularly important strand of this work has focused on integrating principles of embodiment into relationship-centred care, particularly in long-term care settings and for people with advanced dementia, as one strategy for ensuring more inclusive practice that affords people living with severe dementia increased opportunity to exercise citizenship (Kontos et al., 2017).

Although not addressed in all of the literature, it is important to note that there was some emphasis on a person’s sense of self and identity, and how that configures with citizenship. Certainly, citizenship has been connected with the preservation of a person’s identify (Clarke & Bailey, 2016), and with providing a sense of belonging and meaning in one’s life (Baldwin & Greason, 2016; Brannelly, 2016). In addition, Bartlett (2014a, 2014b) has focused on the shared, distinctive and collective identity that can arise for citizens with dementia. What seems clear is that a temporally integrated perspective is necessary to gain a more sophisticated, deeper and nuanced perspective of the full spectrum of citizenship practices across the dementia continuum. In particular, questions regarding how the exercise of citizenship evolves in relation to changes of abilities, life situation, roles, and identities in the dementia trajectory (Bartlett & O’Connor, 2007) emerge as an important area for development. Adopting a more dynamic citizenship approach that takes into account the progressively evolving nature of people’s cognitive capacities due to dementia will enable the delineation of different strategies for different situations, as opposed to a one-size-fits all approach (Nedlund & Nordh, 2015). This kind of tailored approach could be meaningfully developed in future theoretical/conceptual research to better account for the unconditional and total inclusion of people living with dementia as citizens.

From this review, three particularly notable and pertinent areas for future research and conceptualizing emerge. First, although previous research (Bartlett & O’Connor, 2007, 2010; Hulko, 2011; Innes, 2009; O’Connor et al., 2010) has advocated for the need to develop a more intersectional lens for embedding the subjective experience of living with dementia in the broader sociocultural context, to date, with a few exceptions this intersectional lens has not been drawn upon. For example, Bartlett et al. (2018) explored gender but none of the articles explicitly addressed culture, socio-economic status and/or race. Overall, the literature is dominated by a Western view of citizenship. Future research is required that integrates an intersectional lens into perspectives of citizenship of people living with dementia in order to more comprehensively articulate how power associated with people’s differing social locations implicates the exercise of citizenship and rights. This would include exploring how concepts such as autonomy and rights may have decidedly different meanings within the context of more collectivist cultures.

Second, although citizenship implies both rights and responsibilities, to date the focus of scholarship related to citizenship in dementia has focused solely on explicating rights. No attention has been given to understanding citizenship as both rights *and* responsibility. This raises a third point. Whilst there is some recognition that the language of citizenship has facilitated a move forward in conceptualizing how people living with dementia are understood and responded to, there is also a lack of consensus regarding the appropriateness and applicability of a citizenship discourse as the best discourse for moving forward. For example, some attention is being given to the language of vulnerability as a potentially useful reframe for addressing the rights of people living with dementia, whilst simultaneously ensuring that people can access the care and services to which they are entitled (see for example, Hall, 2010; Behuniak, 2010). Recognizing the importance of tying vulnerability to notions of interdependence, future research on the citizenship perspective could more usefully explore ways to resolve the tensions between vulnerability, rights and responsibilities.

## Conclusion

This scoping review has drawn out the different concepts, approaches, and interpretations that shape current understandings of citizenship in the context of dementia practice and research. Thematic exploration of the reviewed literature demonstrate the relationality of citizenship, the nuances of agency, that autonomy can be facilitated, and that there is a strong focus on human rights given a context of stigma and discrimination. These themes are consistent with the definition of citizenship initially proposed by Bartlett and O’Connor (2010) but have been uniquely developed by different researchers. Despite these consistencies, this review does show that citizenship remains to a certain extent an “elusive” concept (Bartlett, 2016), and can be a “malleable and precarious enactment” (Ursin & Lotherington, 2018, p. 1).

Nevertheless, understandings of how the citizenship approach translates into research and practice has begun to expand and the articles covered in this review focus on contexts as varied as long-term care, recreation programs, voting, advocacy, the workplace, and support groups. The adoption of the citizenship approach in dementia studies has important real-world implications and aligns with several policy and practice frameworks, including the PANEL + framework (World Health Organization, 2015), the United Nations (2006) Article 12 of the Convention on the Rights of Persons with Disabilities (CRPD), dementia-friendly communities (DFCs) (Alzheimer’s Disease International, 2016), as well as various national charters (Alzheimer Scotland, 2009; Alzheimer Society of Canada, 2015). Since the concept of citizenship within the context of dementia care, research and practice was first introduced, its strong uptake and consistent use as a pivotal theoretical perspective in dementia studies is apparent given the scope of the literature that this review has uncovered. Despite its potential practical implications, its exploration and use continues to be led by academics. More work is required to actively involve people with dementia in considerations of citizenship, particularly to hear from them what it means to be a citizen living with dementia. Effort should be made to explore intersectional perspectives to reveal understandings of the diversity and heterogenity of citizenship and citizenry within the dementia context. Hard questions need to be asked as to whether current conceptualizations of citizenship adequately capture the requirements and perspectives of people living with dementia, particularly in a world that is increasingly cognizant of the need for recognizing human rights and for eliminating stigma and discrimination.
